# Urethrocutaneous fistulas after voluntary medical male circumcision for HIV prevention—15 African Countries, 2015–2019

**DOI:** 10.1186/s12894-021-00790-y

**Published:** 2021-02-12

**Authors:** Todd Lucas, Jonas Z. Hines, Julia Samuelson, Timothy Hargreave, Stephanie M. Davis, Ian Fellows, Amber Prainito, D. Heather Watts, Valerian Kiggundu, Anne G. Thomas, Onkemetse Conrad Ntsuape, Kunle Dare, Elijah Odoyo-June, Leonard Soo, Likabelo Toti-Mokoteli, Robert Manda, Martin Kapito, Wezi Msungama, James Odek, Jotamo Come, Marcos Canda, Nuno Gaspar, Aupokolo Mekondjo, Brigitte Zemburuka, Collen Bonnecwe, Peter Vranken, Susan Mmbando, Daimon Simbeye, Fredrick Rwegerera, Nafuna Wamai, Shelia Kyobutungi, James Exnobert Zulu, Omega Chituwo, Sinokuthemba Xaba, John Mandisarisa, Carlos Toledo

**Affiliations:** 1 Division of Global HIV and Tuberculosis, Centers for Disease Control and Prevention, Atlanta, GA USA; 2 Global HIV, Hepatitis, and STIs Programmes, World Health Organization, Geneva, Switzerland; 3 Department of Surgery, Edinburgh University, Scotland, UK; 4 Fellows Statistics, Contractor, Centers for Disease Control and Prevention, San Diego, CA USA; 5 U.S. Office of the Global HIV/AIDS Coordinator, Washington, DC USA; 6 Office of HIV/AIDS, U.S. Agency for International Development, Washington, DC USA; 7 Department of Defense, Defense Health Agency, San Diego, CA USA; 8 Ministry of Health and Wellness, Gaborone, Botswana; 9 Division of Global HIV and Tuberculosis, Centers for Disease Control and Prevention, Gaborone, Botswana; 10 Division of Global HIV and Tuberculosis, Centers for Disease Control and Prevention, Nairobi, Kenya; 11 U.S. Agency for International Development, Nairobi, Kenya; 12 Ministry of Health, Maseru, Lesotho; 13 U.S. Agency for International Development, Maseru, Lesotho; 14 Ministry of Health, Lilongwe, Malawi; 15 Division of Global HIV and Tuberculosis, Centers for Disease Control and Prevention, Lilongwe, Malawi; 16 U.S. Agency for International Development, Lilongwe, Malawi; 17 Ministry of Health, Maputo, Mozambique; 18 Division of Global HIV and Tuberculosis, Centers for Disease Control and Prevention, Maputo, Mozambique; 19 U.S. Agency for International Development, Maputo, Mozambique; 20 Ministry of Health and Social Services, Windhoek, Namibia; 21 Division of Global HIV and Tuberculosis, Centers for Disease Control and Prevention, Windhoek, Namibia; 22 National Department of Health, Pretoria, South Africa; 23 Division of Global HIV and Tuberculosis, Centers for Disease Control and Prevention, Pretoria, South Africa; 24 Ministry of Health, Dar es Salaam, Tanzania; 25 Division of Global HIV and Tuberculosis, Centers for Disease Control and Prevention, Dar es Salaam, Tanzania; 26 U.S. Agency for International Development, Dar es Salaam, Tanzania; 27 Division of Global HIV and Tuberculosis, Centers for Disease Control and Prevention, Kampala, Uganda; 28 U.S. Agency for International Development, Kampala, Uganda; 29 Ministry of Health, Lusaka, Zambia; 30 Division of Global HIV and Tuberculosis, Centers for Disease Control and Prevention, Lusaka, Zambia; 31 Ministry of Health and Child Care, Harare, Zimbabwe; 32 Division of Global HIV and Tuberculosis, Centers for Disease Control and Prevention, Harare, Zimbabwe

**Keywords:** Male circumcision, Intraoperative complications, HIV, Fistula

## Abstract

**Background:**

Voluntary medical male circumcision (VMMC) is an HIV prevention strategy recommended to partially protect men from heterosexually acquired HIV. From 2015 to 2019, the President’s Emergency Plan for AIDS Relief (PEPFAR) has supported approximately 14.9 million VMMCs in 15 African countries. Urethrocutaneous fistulas, abnormal openings between the urethra and penile skin through which urine can escape, are rare, severe adverse events (AEs) that can occur with VMMC. This analysis describes fistula cases, identifies possible risks and mechanisms of injury, and offers mitigation actions.

**Methods:**

Demographic and clinical program data were reviewed from all reported fistula cases during 2015 to 2019, descriptive analyses were performed, and an odds ratio was calculated by patient age group.

**Results:**

In total, 41 fistula cases were reported. Median patient age for fistula cases was 11 years and 40/41 (98%) occurred in patients aged < 15 years. Fistulas were more often reported among patients < 15 compared to ≥ 15 years old (0.61 vs. 0.01 fistulas per 100,000 VMMCs, odds ratio 50.9 (95% confidence interval [CI] = 8.6–2060.0)). Median time from VMMC surgery to appearance of fistula was 20 days (interquartile range (IQR) 14–27).

**Conclusions:**

Urethral fistulas were significantly more common in patients under age 15 years. Thinner tissue overlying the urethra in immature genitalia may predispose boys to injury. The delay between procedure and symptom onset of 2–3 weeks indicates partial thickness injury or suture violation of the urethral wall as more likely mechanisms of injury than intra-operative urethral transection. This analysis helped to inform PEPFAR’s recent decision to change VMMC eligibility policy in 2020, raising the minimum age to 15 years.

## Background

Voluntary medical male circumcision (VMMC) decreases the risk of men acquiring HIV through heterosexual sex by approximately 60% [1–4]. The use of VMMC as an HIV prevention measure is a vital component of the Joint United Nations Programme on HIV/AIDS strategy to combat the AIDS epidemic in high-prevalence areas [5]. Since 2009, the President’s Emergency Plan for AIDS Relief (PEPFAR) has supported Ministries of Health (MoHs) and Ministries of Defense to provide over 22.8 million VMMCs in 15 southern and eastern African countries (Botswana, eSwatini, Ethiopia, Kenya, Lesotho, Malawi, Mozambique, Namibia, Rwanda, South Africa, South Sudan, Tanzania, Uganda, Zambia, Zimbabwe) [6].

Moderate and severe adverse events (AEs) occurred in approximately 1–3.5% of VMMC procedures in two of the initial African randomized controlled trials (RCTs), in Uganda and Kenya, while the third such RCT, from South Africa, which did not differentiate among mild, moderate, and severe AEs, reported a rate of 3.8% [1–3]. Rates of moderate and severe AEs from large, established VMMC programs are often reported as much lower in routine program monitoring data; for example, program data report AEs in 0.18% of VMMC procedures in Tanzania and 0.3% for surgical VMMC in Zimbabwe [7, 8]. Although these lower rates may reflect improved competence as a program matures, underreporting of AEs outside the study setting is assumed [9, 10].

A urethrocutaneous fistula (hereafter referred to as fistula) is an abnormal opening between the urethral lumen and the skin that diverts urinary flow from the meatus. In the context of male circumcision (MC), a fistula is an acquired condition resulting from the procedure. These are typically considered the result of damaging the urethral wall by cutting, crushing, or suturing, usually in the region of the frenulum where the urethra is closest to the skin [11–13]. Although post-MC fistulas are very rare, they are a known complication typically documented in case reports or series [12–18]. Case series reported outside a VMMC program setting typically involve fistulas after circumcision done for cultural or religious reasons in the neonatal period or post-neonatal infancy; this complication is often attributed to untrained personnel with poor surgical technique [13, 14, 18]. Concern about this possibility as VMMC programs scaled-up led to inclusion of warnings in the first edition of the World Health Organization’s (WHO) *Manual for Male Circumcision Under Local Anaesthesia* about the risk of urethral injury, particularly when attempting to achieve hemostasis in the frenulum [19].

PEPFAR, through its notifiable adverse event reporting system (NAERS), has conducted routine passive surveillance since 2015 on severe AEs including: deaths, glans injuries, tetanus, AEs resulting in permanent disability (definite or probable), AEs resulting in permanent anatomic deformity (definite or probable), and AEs resulting in hospital admission for ≥ 3 days. Fistulas tend to be reported under one of the last two categories.

As the VMMC program expanded, isolated reports of post-VMMC fistulas began to emerge, mostly in patients under age 15 years. Once a fistula was diagnosed, the patient was referred for management by a specialist and an investigation ensued which then guided prevention activities consisting primarily of provider re-education on proper surgical technique, particularly in young adolescents. PEPFAR and WHO supported implementing partners and ministries in prevention efforts through development of educational materials on fistula prevention, identification, and management, subject matter expert contribution to investigations, and revision of the *Manual for Male Circumcision* to expand on fistula risk factors and mitigation [20–22]. Wide dissemination of these materials was accomplished through both distribution of print materials and webinars.

Despite these efforts, fistula adverse events continue as a rare but persistent complication of VMMC. The primary purpose of this analysis is to describe the collective experience with VMMC-related fistulas in PEPFAR-supported programs and assess the relation of patient age with fistula occurrence. Secondary goals include assessing completeness of the VMMC AE surveillance system to identify gaps in vital information and evaluating current management when fistulas occur. Results will be used to generate hypotheses on risks to guide further prevention efforts.

## Methods

### Data source and collection

All cases of VMMC-associated fistulas reported to PEPFAR through NAERS, from VMMCs performed during 2015–2019 (using U.S. government fiscal year which covers a one year period starting 1 October through 30 September), were retrospectively reviewed. Standardized forms submitted to NAERS contain information gathered during investigation into the procedure, post-operative care, the AE, and its management. A supplemental fistula investigation form, developed by WHO in 2018, was also reviewed when available. This WHO form was developed to improve and standardize data collection on clinical aspects of the case including anatomic variations such as foreskin adhesions, intraoperative events such as frenular bleeding, and provider characteristics including years of experience and number of previous procedures completed.

Data abstraction was performed from the NAERS record to aggregate information including patient demographics, site characteristics, provider and procedure details, fistula management and outcome, and completeness of fistula investigation reporting. Information used in this analysis is routinely collected data from patient charts, clinic registers, and AE investigations.

All patients provided informed written consent for the procedure; consenting for minors was obtained from parents or guardians and adhered to national standards. Data collection and analysis plans were reviewed for ethical considerations in accordance with US Centers for Disease Control and Prevention human research protection procedures.

### Definitions

VMMC facilities were defined as static when routinely performed in a permanent structure, mobile when a team traveled with a consistent moveable structure (e.g. a tent or caravan), and outreach when VMMC teams traveled to on-site permanent or temporary structures and offered services as part of a limited engagement during periods of high demand. All AE types were recorded and classified as described on investigation forms without alteration. For example, if a diagnosis/description of wound infection was not stated, this AE was not assigned even if the patient was given antibiotics.

Initial management was classified as conservative if the patient was not taken urgently for fistula repair upon discovery and had either a catheter placed or at least a month of observation without a catheter between fistula onset and surgical repair. The true management intent of a referral physician was not always clear; this one month time frame was chosen as it implied a lack of urgency for surgical intervention and provided an interval over which improvement may be seen. Although some patients may not have presented immediately after noticing symptoms, the time between fistula onset and presentation was counted towards conservative management because it represented time without surgical intervention. Days from VMMC to fistula was defined as the length of time from the VMMC procedure to the day the patient first noticed symptoms consistent with a fistula (i.e. leakage of urine from a site other than the meatus) or if symptom onset was unknown, when they first presented for care of fistula symptoms. Date of referral is the first documented day the patient saw the referral provider who could offer surgical management of the fistula. Date fistula healed was the earliest date documented stating the fistula was healed.

Conventional surgical circumcisions are typically carried out by one of two methods: dorsal slit (DS) or forceps guided (FG). Due to risk of glans injuries with the FG method, particularly in patients with immature genitalia, WHO advised against use of FG method in prepubertal adolescents and in 2014, PEPFAR mandated use of the DS method in all patients aged 10 to 14 years to minimize the risk of glans injuries.

### Analysis

A descriptive analysis was performed using frequencies for categorical variables and measures of central tendency for continuous variables. Age was dichotomized as < 15 and ≥ 15 years old and rates of fistula AEs were determined using total PEPFAR VMMCs performed in both age groups, in all 15 PEPFAR VMMC countries, and expressed as fistulas per 100,000 VMMCs. The relationship between age group and occurrence of a fistula AE was examined using Fisher’s exact test. Missing data and differences in management led to different denominators for some variables; this is noted where it occurs.

## Results

**Fig. 1:**
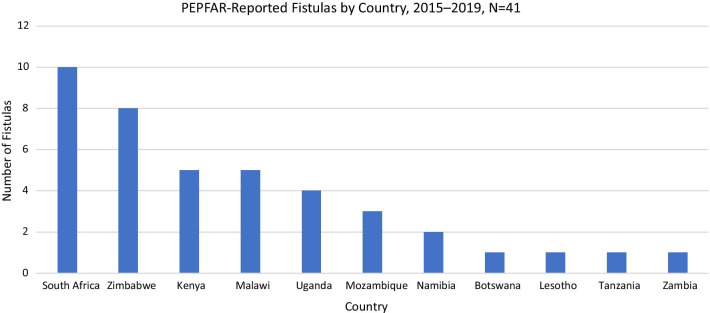
Number of fistulas reported to PEPFAR by country, 2015–2019

From 2015 to 2019, 41 post-VMMC fistulas, from 11 of 15 countries with PEPFAR-supported VMMC programs, were reported (Fig. 1). Complete NAE forms were available for review in 28/41 (68%) of fistula cases. Half of fistula cases (12/24) since 2018 had a WHO supplemental fistula form available. Some data, such as date of VMMC and age of patient, were reliably available and straightforward to collect. Other information, collected from narrative sections of the reporting forms, such as adequacy of provider training, whether a provider was overworked, or if diathermy was used during the procedure, were less often available.

**Fig. 2:**
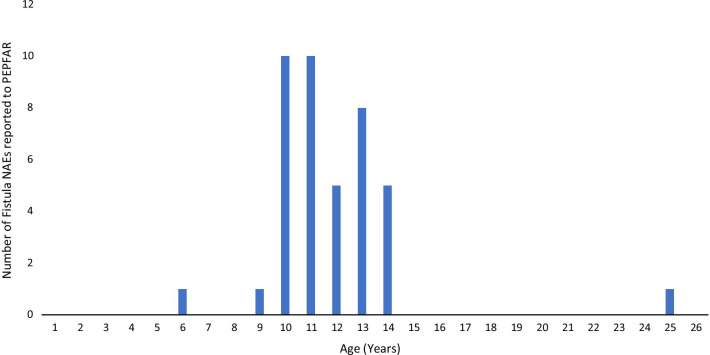
Number of fistula adverse events reported to PEPFAR by age, 2015–2019, N = 41

During 2015 to 2019, approximately 14.9 million PEPFAR-supported VMMCs were performed with an overall rate of 0.28 fistulas per 100,000 procedures. Of the 41 cases, all but one of these fistulas occurred in patients under age 15 years with a median age of 11 years (interquartile range [IQR] 10–13 years old) (Table 1). The greatest number of fistula AEs occurred in patients ages 10 and 11 years (Fig. 2). Though the fistula adverse events were rare, leading to a wide confidence interval, the rate varied considerably by age. The reported rate of events for individuals under age 15 years was 0.61 per 100,000 procedures, while the rate for individuals age 15 years and above was 0.01 per 100,000 procedures. This difference was highly significant (OR = 50.9, [8.6, 2060.0], *p* value < 0.00001) (Table 2).

**Table 1:** Pre- and intra-operative circumstances surrounding fistulas in PEPFAR-supported VMMC programs

	Number (%), N = 41
Patient age in years	
Median (25%, 75%)	11 (10, 13)
Mean (range)	11.8 (6–25)
Modes (n = 10 each)	10, 11
Facility type	
Outreach	21 (51)
Static	15 (37)
Mobile	2 (5)
Unknown	3 (7)
Cadre Type of VMMC provider	
Nurse	7 (17)
Physician	5 (12)
Clinical Officer/ Clinical Associate	3 (7)
Assistant Medical Officer	1 (2)
Unknown	25 (61)
Voluntary medical male circumcision method	
Dorsal slit	35 (85)
Forceps guided	2 (5)
Unknown	4 (10)
Use of diathermy documented	
Yes	6 (15)
No	11 (27)
Not mentioned	24 (59)
Among those completing WHO form (n = 12)	
Frenular bleeding	
Yes	1 (8)
No	11 (92)
Length of MC provider experience	
6 months	3 (25)
About a year	2 (17)
About 2 years	2 (17)
3 years or more	5 (42)
Number of VMMCs done by provider	
Only a few	0
More than 10	2 (17)
More than 50	4 (33)
More than 100	6 (50)
Anatomic condition documented (e.g. adhesions, tight frenulum)	
Yes	3 (25)
No	9 (75)
Patients had at least one follow-up visit within 7 days of MC	
Yes	34 (83)
No	5 (12)
Unknown	1 (2)
Not applicable*	1 (2)

^*^Patient had glans injury during MC and was immediately transferred for management

**Table 2:** Odds ratio of fistula occurrence in patients under age 15 years compared to those age 15 years and older in PEPFAR-supported VMMC programs, 2015–2019

	Fistula	No fistula	Odds ratio*	95% CI	Reported rate of fistula occurrence
< 15 years old	40	6,546,926	50.9	8.6–2060.0	0.61 per 100,000 VMMC
≥ 15 years old	1	8,329,144	-	–	0.01 per 100,000 VMMC

^*^Fisher’s Exact Test, *p* value < .00001

Fistula cases occurred in all types of facilities with slightly over half of involved facilities (21/41, 51%) identified as outreach sites (Table 1). The type of provider was reported for 16 of the 41 cases. Among the cases where type of provider was reported, both physician (5/16, 31%) and various types of non-physician providers (11/16, 69%) performed circumcisions that resulted in fistulas. Of the 12 cases where a WHO fistula investigation form was available, providers reported a wide range of both years of VMMC experience and number of previous VMMC procedures. Among the 17 fistula cases where the records commented on diathermy use, 6 (35%) documented its use. Most cases (35/41, 85%) had their circumcision performed using the DS method and 34/41 (83%) presented for scheduled follow-up within 7 post-operative days.

The median time from VMMC to appearance of fistula symptoms was 20 days (IQR 14–27 days) (Table 3). Only 5/41 (12%) fistulas appeared within the first week after surgery. Most patients (31/41, 76%) had fistula as their initial AE diagnosis, with infection being the second most common initial diagnosis (7/41, 17%). Of the 28 cases with another type of post-VMMC AE diagnosed, 21/28 (75%) were infections. Concerning management once a fistula was diagnosed, most patients (40/41, 98%) were documented as having seen a referral specialist for fistula management where all but 2 had a documented specialty. Urologists were the most common referral specialty (29/38, 76%). Once fistula symptoms started, patients were seen by referral specialists in a median of 6 days (IQR 2–19 days) with 38/41 (93%) documenting an initial attempt at conservative management of which 4/38 (11%) had documented success, 10/38 (26%) had documented failure, and 27/38 (71%) had an unknown outcome. Nine of the total 41 patients (22%), all of whom underwent initial conservative management, had at least one attempt at surgical repair with 4/9 (44%) having documented success. Of the 9 patients with known referral and surgical dates, the median time from fistula appearance to undergoing initial surgical repair was 60 days (IQR 30–74 days). There were 40 patients with known follow-up dates who were followed for a median of 38 days after fistula onset (IQR 15–100) and in 30/41 (73%), the fistula remained unhealed on the date of last documentation.

**Table 3:** Post-operative circumstances surrounding fistulas in PEPFAR-supported programs

	Number (%), N = 41
Days from VMMC to fistula symptoms	
Median (25%, 75%)	20 (14, 27)
Mean (range)	30 (3–183)
Initial AE type diagnosed	
Fistula	31 (76)
Infection	7 (17)
Urinary retention	1 (2)
Glans injury	1 (2)
Wound	1 (2)
Other AE (was any other AE diagnosed in addition to fistula)	
Yes	28 (68)
Infection (% of n = 28)	21 (75)
Stricture (% of n = 28)	4 (14)
Wound/dehiscence (% of n = 28)	1 (4)
Stricture and wound (% of n = 28)	1 (4)
Glans injury (% of n = 28)	1 (4)
No	13 (32)
Referred	
Yes	40 (98)
No	0
Unknown	1 (2)
Specialty of referral provider	
Urologist	29 (71)
Pediatric Surgeon	4 (10)
Plastic Surgeon	4 (10)
Other Surgeon	1 (2)
Unknown Referral Provider Type	2 (5)
Unknown if referred	1 (2)
Days from fistula appearance to referral (if referral date known) (n = 35^†^)	
Median (25%, 75%)	6 (2, 19)
Mean (range)	15 (0–75)
Antibiotic treatment	
Yes	32 (78)
No	9 (22)
Initial Management	
Conservative	38 (93)
Surgical	0
Unknown	3 (7)
Conservative management outcome (n = 38)	
Successful (% of n = 38)	4 (11)
Unsuccessful	10 (26)
Unknown	27 (71)
Ever had surgical repair	
Yes	9 (22)
No	4 (10)
Unknown	28 (68)
Days from fistula appearance to surgical repair (n = 9)	
Median (25%, 75%)	60 (30, 74)
Mean (range)	57 (16–103)
If surgery documented, what was the outcome (n = 9)	
Successful (% of n = 9)	4 (44)
Unsuccessful	3 (33)
Unknown	2 (22)
If surgery performed, total number of operations documented (n = 9)	
One (% of n = 9)	8 (89)
Two	1 (11)
Fistula healed at last documented follow up	
Yes	9 (22)
No	30 (73)
Unknown^‡^	2 (5)

^†^Outlier of 213 days excluded

^‡^Two with corrective surgery performed but no follow-up results documented

## Discussion

During 2015–2019, PEPFAR-supported programs reported 41 VMMC-related fistulas. Although these are exceedingly rare AEs, they can be very difficult to manage and often require repeated attempts at surgical repair [14]. Despite numerous attempts to prevent fistulas through provider education and training, these AEs continue to occur, and further interventions are needed.

In this analysis, age under 15 years was associated with the occurrence of a VMMC related fistula. The reported rate of fistula occurrence in < 15 year olds is similar to that found for glans injuries in this same age group [23]. This finding is further supported by the biologic plausibility of a younger patient sustaining injury to less mature (smaller) genitalia during VMMC. With thinner tissue between the urethra and skin, especially at the frenulum, these patients are more prone to urethral injury with variations in surgical technique. For example, if a provider places a deep hemostatic suture in the frenulum of an adult patient, the comparatively thicker tissue may accommodate it; whereas, in an immature penis, a suture placed at the same depth could end up in the urethral lumen. Likewise, variations in contact time during application of diathermy, or method of skin suture placement, could be harmless in an adult but risk urethral injury in a young adolescent. Although other case series have noted a preponderance of post-circumcision fistulas in children, it is unclear how many adult males were being circumcised to allow comparison [13, 14, 16, 18]. Since VMMC programs for HIV prevention circumcise more older adolescents and adult males, this provides an opportunity to compare these two age groups.

Although the DS method of circumcision was most commonly seen in this analysis, this is likely related to this method being recommended for use in patients < 15 years of age during VMMCs due to the risk of glans injury with the alternative FG method [23]. Use of the FG method could also be under-documented due to fear of punishment for using it against policy in patients < 15 years old.

The median interval from surgery to fistula appearance of 14–27 days indicates the mechanism of injury is unlikely to be a direct cut into the urethra during the procedure since this should be evident much sooner, certainly within a day or two of surgery. However, polyglactin sutures, commonly used in VMMC programs, are typically absorbed by the body in this timeframe, and therefore if a skin or hemostatic suture is placed through the full thickness of the urethral wall, this violation may not be evident until the suture material dissolves. Ischemic injuries of the urethral wall, either from improper diathermy technique or incorporation of the wall in a suture, would also likely stay intact until tissue necrosis and sloughing takes place. All cadre types and levels of provider experience were involved with fistulas, but report forms did not collect potentially relevant information such as amount of provider experience specifically with young adolescent patients, provider workload for the day, previous fistula prevention training, adequacy of anesthesia, time of day, and adequacy of operative field lighting. Even an experienced provider could have suboptimal technique in the setting of inadequate lighting or a moving patient due to inadequate anesthesia.

Infection leading to a fistula has been reported [24]. However, most of the concurrent diagnoses of wound infection in this analysis developed after the fistula symptoms had started, which would argue against infection as an inciting event. Even in the cases where a wound infection is diagnosed first, risk factors for both, such as a large amount of ischemic tissue from excessive diathermy or hemostatic sutures, could be present as a common root cause.

Fistulas alone, although highly concerning to the patient, do not require urgent intervention. They should, however, be referred to an experienced physician who is competent to perform a surgical repair. It is reassuring that patients in this analysis were seen by specialists, typically urologists, usually within a week of symptom appearance. A full discussion on appropriate management of fistulas is beyond the scope of this paper but important components of care typically involve a period of at least 3–6 months of conservative management to allow the wound time to stabilize; this may consist of time alone or may include additional measures such as placement of a urinary catheter, wound care, or antibiotics [14, 18, 25]. The median time of 60 days from fistula appearance to repair in this analysis was less than the 3–6 months recommended. Waiting longer before attempting repair represents an area for improvement in the observed fistula management. Although most final management outcomes are unknown, there were four instances of complete healing without surgery. Given the complexity of urethral reconstructions, and the increased difficulty with subsequent repairs, the first attempt should be performed by a specialist with experience in fistula repair [13, 14, 25].

Despite multiple interventions aimed at fistula prevention, primarily involving provider education and training, these rare but devastating AEs have continued to occur. Unlike glans amputations, which result from a particular surgical method that can be completely avoided, fistulas likely result from subtle variations during surgical maneuvers that cannot be avoided when performing a surgical circumcision such as sharp dissection, hemostasis, and suturing. Additional potential contributing factors such as inadequate lighting, use of diathermy in the frenulum, large needles that encourage deeper bites, surgical instruments of inappropriate size, patients who are unable to lie still, and rushing through a procedure resulting in decreased surgical precision, were identified by subject matter experts attending a recent WHO-sponsored meeting focusing on VMMC-related fistulas [25].

Strengths of this analysis include the large volume of both procedures and fistulas, standardized reporting of case investigations to allow aggregation and comparison, and wide representation of countries across the PEPFAR-supported program. This retrospective analysis includes the risk of bias due to unknown systematic errors in reporting or classification of fistulas and the risk of unknown confounding of the association of age with fistula especially given incomplete reporting forms and missing information about potential contributing risks. Some important areas where additional information would be valuable include: provider training, provider workload, adequacy of operative field lighting, use of diathermy, components of conservative management, and outcomes. Another limitation is, due to the voluntary nature of the passive reporting system, providers may have been reluctant to report fistulas due to fear of personal repercussions. While underreporting of fistulas is possible, it is unknown if systematic differences occur between age groups, or across geographic units. Given this, comparison between countries based on these data is not recommended. Furthermore, providers may underreport fistulas because the NAERS form requires them to make a judgement call about whether a fistula will result in permanent deformity or disability. Finally, aside from the increased association with age, findings from this analysis should be viewed as hypothesis-generating due to lack of comparison with controls.

## Conclusions

Although improvements to any identified deficiencies at the program, facility, or provider levels can be helpful, interventions tend to be more effective the further they are from reliance on the actions of any one individual [26]. An example of this would be to avoid open surgical circumcisions in patients under age 15, the decision recently made by PEPFAR and supported by this analysis [27]. Although there is high demand among 10–14 year olds in many countries, waiting until they have matured and aged out of this high-risk group should retain the same HIV-prevention benefits while improving patient safety. The use of circumcision devices, instead of open surgical methods, in younger patients may not confer the same risk for a fistula however given the rarity of these complications, more data are needed to allow comparison. Additional recommendations including avoiding diathermy use in the frenulum, changing to a smaller needle size (19 mm instead of the typical 26 mm) [27], adequate operative field lighting, on-going monitoring and evaluation of service quality, and ensuring an adequate amount of trained staff for patient volume will likely provide improvements to safety regardless of patient age. Finally, when a fistula does occur, referral to a surgeon experienced in urethral reconstruction for all further management is needed for an optimal outcome.

## Data Availability

The dataset used during the present study is available from the corresponding author on reasonable request.

## Abbreviations

VMMC: Voluntary medical male circumcision
PEPFAR: President’s Emergency Plan for AIDS Relief
AE: Adverse event
MoH: Ministry of Health
RCT: Randomized Controlled Trial
MC: Male circumcision
WHO: World Health Organization
NAERS: Notifiable Adverse Event Reporting System
DS: Dorsal slit
FG: Forceps-guided
